# E‐cadherin expression is associated with somatostatin analogue response in acromegaly

**DOI:** 10.1111/jcmm.13851

**Published:** 2019-03-06

**Authors:** Eva Venegas‐Moreno, Alvaro Flores‐Martinez, Elena Dios, Mari C. Vazquez‐Borrego, Alejandro Ibañez‐Costa, Ainara Madrazo‐Atutxa, Miguel A. Japón, Justo P. Castaño, Raúl M. Luque, David A. Cano, Alfonso Soto‐Moreno

**Affiliations:** ^1^ Unidad de Gestión de Endocrinología y Nutrición Instituto de Biomedicina de Sevilla (IBiS) Hospital Universitario Virgen del Rocío/CSIC/Universidad de Sevilla Sevilla Spain; ^2^ Instituto Maimónides de Investigación Biomédica de Córdoba (IMIBIC) Córdoba Spain; ^3^ Department of Cell Biology, Physiology and Immunology Universidad de Córdoba Córdoba Spain; ^4^ Hospital Universitario Reina Sofía Córdoba Spain; ^5^ CIBER Fisiopatología de la Obesidad y Nutrición (CIBERObn) Córdoba Spain; ^6^ Instituto de Biomedicina de Sevilla (IBIS) Hospital Universitario Virgen del Rocío/CSIC/Universidad de Sevilla Sevilla Spain; ^7^ Department of Pathology Hospital Universitario Virgen del Rocío Sevilla Spain

**Keywords:** acromegaly, E‐cadherin, pituitary tumour, somatostatin analogues, somatostatin receptor

## Abstract

Acromegaly is a rare disease resulting from hypersecretion of growth hormone (GH) and insulin‐like growth factor 1 (IGF1) typically caused by pituitary adenomas, which is associated with increased mortality and morbidity. Somatostatin analogues (SSAs) represent the primary medical therapy for acromegaly and are currently used as first‐line treatment or as second‐line therapy after unsuccessful pituitary surgery. However, a considerable proportion of patients do not adequately respond to SSAs treatment, and therefore, there is an urgent need to identify biomarkers predictors of response to SSAs. The aim of this study was to examine E‐cadherin expression by immunohistochemistry in fifty‐five GH‐producing pituitary tumours and determine the potential association with response to SSAs as well as other clinical and histopathological features. Acromegaly patients with tumours expressing low E‐cadherin levels exhibit a worse response to SSAs. E‐cadherin levels are associated with GH‐producing tumour histological subtypes. Our results indicate that the immunohistochemical detection of E‐cadherin might be useful in categorizing acromegaly patients based on the response to SSAs.

## 1 INTRODUCTION

Acromegaly is a rare disease resulting from hypersecretion of growth hormone (GH) and concomitant insulin‐like growth factor 1 (IGF1) typically caused by pituitary adenomas termed somatotropinomas. GH and IGF1 excess are associated with increased mortality and morbidity,^1^ and thus, the reduction in GH and IGF1 levels is considered the main therapeutic goal in acromegaly.

Consensus guidelines recommend somatostatin analogues (SSAs) as the therapy of choice for pharmacological treatment of acromegaly either as adjuvant therapy in patients after unsuccessful pituitary surgery or when surgery is considered not feasible.^2^ However, the response to SSAs treatment is largely variable.^3^, ^4^, ^5^ Recent prospective studies have shown success rates for SSAs (20%‐40% of patients) lower than initially reported (recently reviewed in^6^). While differences in patient selection and definitions of response to treatment may partly account for these discrepancies among published studies, there is certainly considerable variability in the efficacy of SSAs among patients in each individual study. Thus, the discovery of the factors involved in resistance to SSAs and/or in predicting patient response to SSAs treatment might help to individualize therapeutic treatments in acromegaly patients.

A number of histopathological and molecular markers of response to SSAs have been proposed during the last decades but none has been incorporated into routine clinical practice or in clinical guidelines for the management of acromegaly patients. Molecular markers such as AIP, ZAC1 and RKIP and, prominently, somatotastin receptor subtypes (SSTRs) has been analysed in GH‐producing pituitary adenomas at the mRNA or protein level.^7^, ^8^, ^9^, ^10^, ^11^, ^12^, ^13^ Another molecular marker associated with SSAs response is the accumulation of E‐cadherin.^14^ E‐cadherin is a cell adhesion protein located at the cytoplasmic membrane and reported to work as a tumour suppressor. Loss of E‐cadherin expression is associated with increased invasive and metastatic ability in a variety of tumours such as breast and lung tumours.^15^, ^16^ The link between loss of E‐cadherin and invasive tumour behavior might be related to the induction of epithelial‐to‐mesenchymal transition (EMT) commonly observed in the most advanced phases of these tumours. Thus, E‐cadherin down‐regulation is considered a hallmark of EMT. Decreased expression of E‐cadherin in pituitary (including GH‐producing) adenomas has been previously reported.^14^, ^17^, ^18^, ^19^, ^20^ However, the association between the loss of E‐cadherin expression and aggressiveness of GH‐producing tumours has yielded conflicting results.^14^, ^17^, ^18^, ^19^, ^20^ E‐cadherin expression levels are also correlated with GH‐producing tumour histological subtypes. Thus, whereas high E‐cadherin expression levels are found in densely granulated somatotroph adenomas (DGSAs) tumours, low or absent E‐cadherin expression is observed in sparsely granulated somatotroph adenomas (SGSAs).^21^, ^22^, ^23^, ^24^, ^25^ Of note, the granulation pattern of GH‐producing tumours is considered a histological marker of response to SSAs treatment with SG somatotropinomas presenting a worse response to SSAs treatment.^26^ Despite all these findings, the potential association between E‐cadherin expression and SSAs response in GH‐producing tumours has been barely studied to date.^14^ Fougner et al^14^ reported that loss of membranous E‐cadherin expression and concomitant translocation of E‐cadherin to the nucleus was associated with resistance to SSAs treatment in GH‐producing tumours. In this study, E‐cadherin expression was assessed by immunohistochemistry (IHC) using two different antibodies directed against either the intracellular or the extracellular domain of the protein. Importantly, their results revealed that a significant association between E‐cadherin expression and response to SSAs could only be found when E‐cadherin accumulation was evaluated with the intracellular domain antibody.^14^ Therefore, the choice of antibody may severely influence the potential predictive value of E‐cadherin accumulation for the SSAs treatment response in acromegaly patients.

Here, we performed a precise histological and immunohistochemical E‐cadherin examination in GH‐producing pituitary tumours using an automated system and an E‐cadherin antibody widely used in diagnostic pathology. Our aim was to identify the potential association between the response to SSAs treatment and E‐cadherin expression. Moreover, we analysed the relationship between E‐cadherin expression and GH‐producing histological subtypes as well as SSTRs expression.

## 2 MATERIAL AND METHODS

### 2.1 Patients and samples

The study population consisted of 55 acromegaly patients who were evaluated retrospectively and identified from a series of 152 acromegaly patients who underwent transsphenoidal surgery in the Virgen del Rocío University Hospital between 1998 and 2014.^13^ The diagnosis was based on clinical and biochemical features and confirmed immunohistochemically by an experienced pathologist. Fifty‐five patients whose archival tissue was available and of enough quality for IHC were included. These 55 patients have been described in a previous study comprising a larger cohort of acromegaly patients.^13^ The usual clinical practice in our hospital is that all acromegaly patients are treated with SSAs (octreotide or lanreotide) while waiting for surgery^27^ regardless of their responsiveness to SSAs. Thus, all acromegaly patients included in this study have been preoperatively treated with SSAs. Indeed, seven patients were excluded for this study because of either lack of preoperative treatment or preoperative treatment with dopamine agonists (three and four patients, respectively). Patients were treated with SSAs until the day before surgery. After surgery, patients remained without SSAs treatment until evaluation for surgical remission, performed at least 3 months after surgery following acromegaly guidelines.^2^ If patients were considered not cured based on clinical and biochemical data, SSAs treatment was resumed. No patient received radiotherapy before surgery. Of the 55 patients, it was possible to obtain reliable biochemical data to evaluate the response to SSAs treatment from 41 patients either before surgery (27) or as adjuvant after unsuccessful surgery (14). Missing data were because of incomplete follow‐up. Twenty‐eight patients were treated with octreotide long‐acting release (30 mg) and 13 with lanreotide autogel (120 mg). Responsiveness to SSAs was assessed by per cent IGF1 reduction after 3 and 6 months of treatment from the time of diagnosis (preoperative therapy) or from the time of surgical failure evaluation (adjuvant therapy). An IGF1 per cent reduction higher than 50% was considered positive response. Disease control was also assessed according to consensus criteria.^28^

Percentages above the upper limit of normal (%ULN) for age‐ and gender‐matched IGF1 levels were calculated. Tumour size and cavernous sinus invasion data were obtained from magnetic resonance images. Cavernous sinus invasion was evaluated using the Knosp classification.^29^ Knosp grade 3 and 4 were defined as invasive. For RNA extraction, a piece of the pituitary tumour was immediately frozen after surgery removal on dry ice and stored at −80**°**C until assayed. This study was conducted following the ethical standards of the Helsinki Declaration of the World Medical Association and approved by the IBiS‐Virgen del Rocio Hospital Ethics Committee. Written informed consent was obtained from each participant or relative in case of autopsy.

### 2.2 Histopathology and immunohistochemistry

The construction of the tissue microarray (TMA) containing formalin‐fixed paraffin‐embedded tissues from 55 GH‐secreting pituitary adenomas has been previously reported.^13^ Normal pituitary tissue included in the TMA was obtained from the HUVR‐IBiS BioBank. GH‐producing histological subtypes were identified using cytokeratin CAM5.2 immunostaining (Cell Marque, Sigma, Madrid, Spain) with an automated immunostainer system (Ventana Medical systems, Roche, Basel, Switzerland) and histological characteristics. DGSAs were defined by immunostaining of CAM5.2 in a diffuse perinuclear pattern in more than 70% of tumour cells. SGSAs were defined as paranuclear, spherical pattern of CAM5.2 in more than 70% of tumour cells. SGSAs usually exhibit weaker GH immunoreactivity than DGSAs. Immunohistochemical analysis for E‐cadherin was performed using an E‐cadherin mouse monoclonal antibody directed against the intracellular domain of the protein (ready‐to‐use, clone 36, VENTANA, Roche, catalogue number 790‐4497) with an automated immunostainer system (VENTANA, Roche) following the manufacturer's specifications. The adenomas were assessed in a semiquantitatively scored blindly by two researchers and classified on a three‐tier scale from 1 to 3: score 1, no or extremely low immunoreactivity; score 2, mild to moderate membranous accumulation (immunoreactivity in <50% of tumour cells); and score 3, extensive membranous accumulation (immunoreactivity in more than 50% of tumour cells). This score system is similar to that used for SSTR scoring.^13^, ^30^ Bright‐field images were captured using a BX‐61 microscope (Olympus, Madrid, Spain).

### 2.3 RNA isolation, reverse transcription and analysis of gene expression by quantitative real‐time PCR

Somatostatin receptor (SSTR1‐SSTR5) and dopamine receptor (DRD1‐DRD5) expression by quantitative real‐time PCR (qPCR) in the 55 patients included in this study have been previously analysed.^13^ Technical details on RNA extraction, reverse‐transcription and qPCR quantification have been described elsewhere.^31^, ^32^, ^33^ Gene expression values were normalized to beta‐actin mRNA levels. We have found beta actin to be a housekeeping gene with stable expression in pituitary adenomas, as described in previous studies from our group.^13^, ^32^, ^34^

### 2.4 Statistical analysis

Normality of the data was tested using the Kolmogorov‐Smirnov test. The categorical variables are described as percentages and frequencies. Normally distributed data are presented as means ± SD unless noted otherwise. For non‐normally distributed data, median values with interquartile ranges (IQR) are shown. Data were analysed using Mann‐Whitney and Kruskal‐Wallis test for nonparametric variables and ANOVA and Student's *t* test for parametric variables. For categorical variables, chi‐square was used. Statistical analysis was performed using SPSS software version 23.0 for Windows (SPSS, Chicago, IL, USA). *P* values <0.05 were considered statistically significant.

## 3 RESULTS

### 3.1 Patient and sample characteristics

A total of 55 GH‐producing tumours from acromegaly patients were studied. The baseline clinical characteristics of the study population are shown in Table 1. All patients underwent transsphenoidal surgery. Forty‐seven (85.4%) tumours were macroadenomas. Ten (18.2%) of the adenomas displayed both GH and PRL expression while the remaining 45 were pure GH‐producing tumours.

**Table 1:** Baseline characteristics of the study cohort

Characteristics	
Sex (% female)	52.7%
Age at diagnosis (years, median, IQR)	39 (32‐47)
Maximum tumour diameter at diagnosis (mm, median, IQR)	20 (12.8‐29)
GH at diagnosis (ng/mL, median, IQR)	21.4 (8‐40)
IGF1 at diagnosis (% ULN, median, IQR)	260.3 (202.8‐311.1)

Data are presented as median with interquartile ranges (IQR). ULN, upper limit of normal for age‐ and gender‐matched IGF1 levels.

### 3.2 E‐cadherin expression assessed by IHC in GH‐secreting adenomas

**Figure 1:**
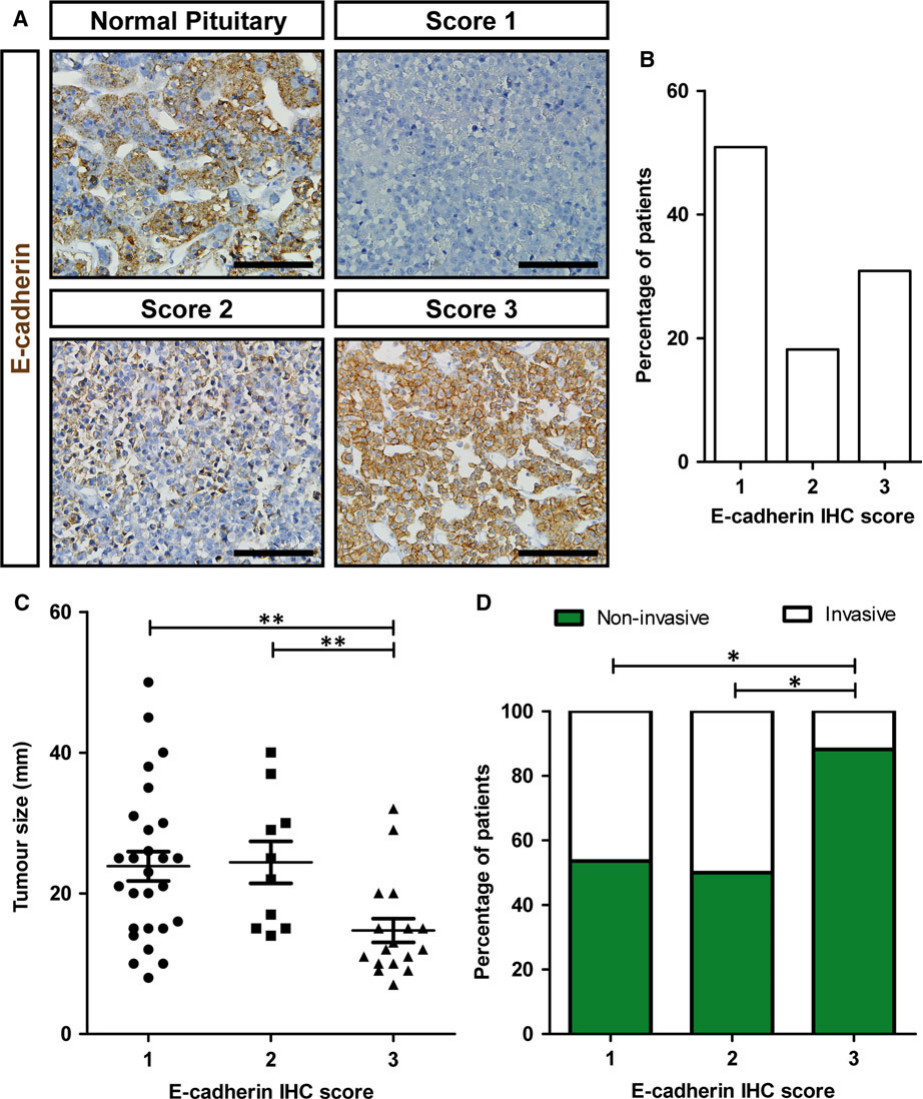
Immunohistochemical detection of E‐cadherin in GH‐producing tumours. A, Representative images of E‐cadherin immunohistochemical (IHC) scores in normal human pituitary and somatotropinomas. Score 1, no or very low immunoreactivity; score 2, membranous immunoreactivity in <50% of cells; score 3, membranous immunoreactivity in more than 50% of cells. Scale bar: 100 μm. B, Percentage of somatotropinomas for each E‐cadherin IHC score. C, Comparison of tumour size with the different E‐cadherin IHC scores. Data points represent values for each individual patient. Mean and SEM are also displayed. The Kruskal‐Wallis test was used for comparison among the three scores and the Mann‐Whitney test for post hoc comparisons. D, Percentage of invasive tumours compared to E‐cadherin IHC score. The chi‐square test was used. *P < 0.05; **P < 0.01

Robust membranous E‐cadherin staining was observed in normal human pituitary (Figure 1A). However, not all pituitary cells were positive for E‐cadherin, in agreement with previous studies.^20^ Representative images of E‐cadherin immunoreactivity in normal pituitary and the different IHC semiquantitative scores in somatotropinomas are shown in Figure 1A. Twenty‐eight tumours displayed none or extremely low, negligible membranous staining. Ten tumours displayed mild to moderate membranous immunoreactivity (<50% of the tumour cells). The remaining 17 tumours displayed strong membranous immunoreactivity in more than 50% of the cells (Figure 1B). No nuclear immunoreactivity was observed in any of the pituitary tumours.

### 3.3 Association between E‐cadherin expression and baseline biochemical and clinical characteristics

At baseline, tumour size was significantly different among the three E‐cadherin IHC scores (*P* = 0.003), namely it was lower in the score 3 than in scores 2 and 1 (Figure 1C). The median tumour size for score 1 was 23 mm (IQR, 15‐30), 23.5 mm (IQR, 15‐31.8) for score 2 and 12 (IQR, 10‐17.5) for score 3. Tumours with score 3 were less likely to be invasive than tumours with score 2 or 1 (Figure 1D). We did not find statistically significant differences in sex, age and GH or IGF1 levels (assessed by per cent increase from upper limit of normal) among the three different E‐cadherin IHC scores.

### 3.4 Association between response to somatostatin analogue treatment and E‐cadherin expression

Clinical data to conclusively establish the response to SSAs were available for 41 patients at 3 months of treatment (27 before surgery and 14 as adjuvant therapy) and for 36 patients after 6 months of treatment (19 before surgery and 17 as adjuvant therapy). As no differences in the response to SSAs between patients treated preoperatively or as adjuvant therapy (both at 3 and 6 months after treatment) were observed, we decided to analyse all the response data as one single group.

**Figure 2:**
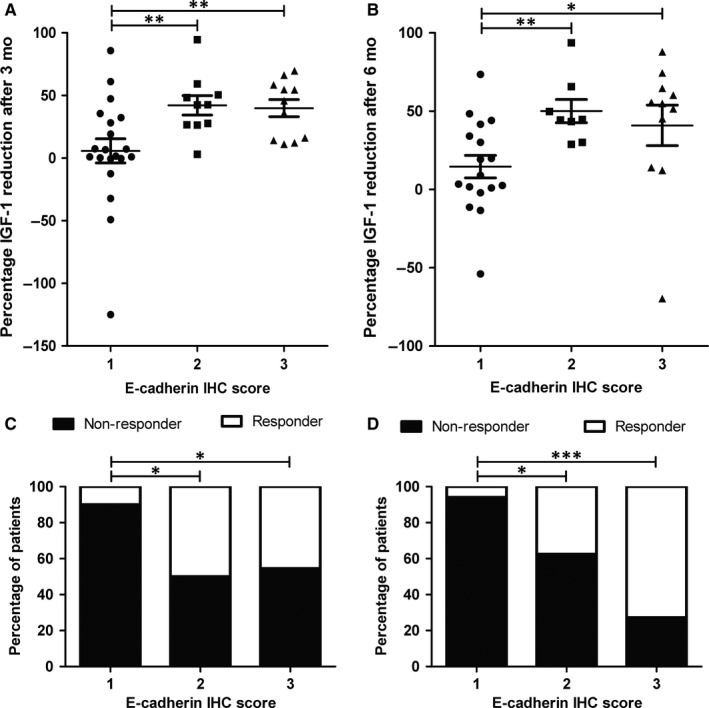
Insulin‐like growth factor 1 (IGF1) per cent reduction after somatostatin analogues (SSAs) treatment and E‐cadherin score. A, Comparison of IGF1 per cent reduction after 3 mo of SSAs treatment with the different E‐cadherin immunohistochemistry (IHC) scores. B, Comparison of IGF1 per cent reduction after 6 mo of SSAs treatment with the different E‐cadherin IHC scores. Data points represent values for each individual patient. Mean and SEM are also displayed. The Kruskal‐Wallis test was used for comparison among the three scores and the Mann‐Whitney test for post hoc comparisons. C, Percentage of patients responsive to SSAs treatment after 3 mo compared to E‐cadherin IHC score. The chi‐square test was used. D, Percentage of patients responsive to SSAs treatment after 6 mo compared to E‐cadherin IHC score. The chi‐square test was used. *P < 0.05; **P < 0.01; ***P < 0.001

Median IGF1 per cent reduction at 3 and 6 months was 26.5% (IQR, 2.3‐49.3) and 37.9% (IQR, 4.7‐53.9), respectively. Twelve patients responded to SSAs (IGF1 per cent reduction higher than 50%) at three (29.3%) and six (33.3%) months, respectively. No differences were observed regarding age, sex, tumour size and GH and IGF1 levels at diagnosis between patient responders and non‐responders either at 3 or 6 months after treatment. In contrast, a marked difference in IGF1 per cent reduction after SSAs treatment was found at both 3 and 6 months of treatment among the three E‐cadherin IHC scores (*P* = 0.004 and 0.006, respectively) (Figure 2A,B). Specifically, a lower IGF1 per cent reduction was observed at 3 and 6 months in the score 1 compared to scores 2 and 3 (*P* = 0.008 and 0.005, for 3 and *P* = 0.006 and 0.012, for 6 months, respectively) (Figure 2A,B). No differences in IGF1 per cent reduction were found between scores 2 and 3, either at 3 or 6 months after treatment (Figure 2A,B). At 3 months of treatment, the median IGF1 per cent reduction for score 1 was 4.1 (IQR, −0.5 to 31.2), 42.5 (IQR, 26.5‐52.6) for score 2, and 45.8 (IQR, 14‐58.4) for score 3. At 6 months of treatment, the median IGF1 per cent reduction for score 1 was 8.9 (−0.6 to 37.9), 44.5 (IQR, 33.4‐61.7) for score 2, and 54.8 (IQR, 14‐64.5) for score 3. Only two of the patients with tumours with score 1 (out of 20) were responders at 3 months (Figure 2C) and only one tumour was responder at 6 months (Figure 2D). At 3 months of treatment, 50% of adenomas with a score of 2% and 45.5% of adenomas with a score of 3 were considered responders (Figure 2C). At 6 months of treatment, 37.5% of adenomas with a score of 2% and 72.7% of adenomas with a score of 3 were considered responders (Figure 2D). Disease control^28^ by SSAs treatment at 6 months was achieved in 33.3% of patients (12 out of 36). Only two of the patients with tumours with score 1 (out of 17), while 5 patients (out of 8; 62.5%) classified as score 2, and 5 (out of 11; 45.5%) classified as score 3 achieved disease control (chi‐square test, *P* = 0.025).

Importantly, there was no difference in the duration of preoperative SSA treatment between responder (8, IQR, 3‐11.3 at 3 months and 8.5, IQR, 6.3‐13.5 at 6 months of treatment) and non‐responder patients (6, IQR, 3‐10.5 at 3 months and 5.5, IQR, 2.3‐10.6 at 6 months of treatment) (*P* = 0.57 and 0.22 at 3 and 6 months, respectively) that could have potentially affected E‐cadherin expression.

### 3.5 Relationship between E‐cadherin and dopamine and somatostatin receptor expression

We have previously described an association between the response to SSAs treatment and the expression of SSTR1 and SSTR2 as well as DRD4 and DRD5, as assessed by qPCR.^13^ However, no difference in the gene expression levels of these receptors (or, for that matter, any other measured SSTRs and DRDs) was found among the different E‐cadherin IHC scores (data not shown). We also analysed the potential association between E‐cadherin expression and SSTR protein levels as evaluated by IHC. Again, no association was found between E‐cadherin IHC scores and SSTR2 and SSTR3 IHC scores (*P* = 0.22 and 0.79, respectively). However, an intriguing inverse relationship was found between E‐cadherin IHC scores and SSTR5 grading scores (*P* = 0.012). GH‐producing tumours with E‐cadherin score of 1 were more likely to have a SSTR5 IHC score of 3 and less likely to have a SSTR5 IHC score of 1 (Table 2). Conversely, tumours with E‐cadherin score of 3 were less likely to have a SSTR5 IHC score of 3 and more likely to have a SSTR5 IHC score of 1 (Table 2).

**Table 2:** Association of E‐cadherin and SSTR5 immunohistochemistry scores

E‐cadherin IHC score	SSTR5 IHC score		
	1	2	3
1	5^a^	8	15^b^
2	3	4	3
3	10^b^	6	1^a^

a Lower frequency with respect to the other IHC scores in the adjusted residual analysis (residual was smaller than −1.96, indicating that the number of cases in that cell is significantly smaller, with a significance level of *P* = 0.05).

b Higher frequency with respect to the other IHC scores in the adjusted residual analysis (residual was higher than 1.96, indicating that the number of cases in that cell is significantly larger, with a significance level of *P* = 0.05).

### 3.6 Association between adenoma granulation pattern and E‐cadherin expression

**Figure 3:**
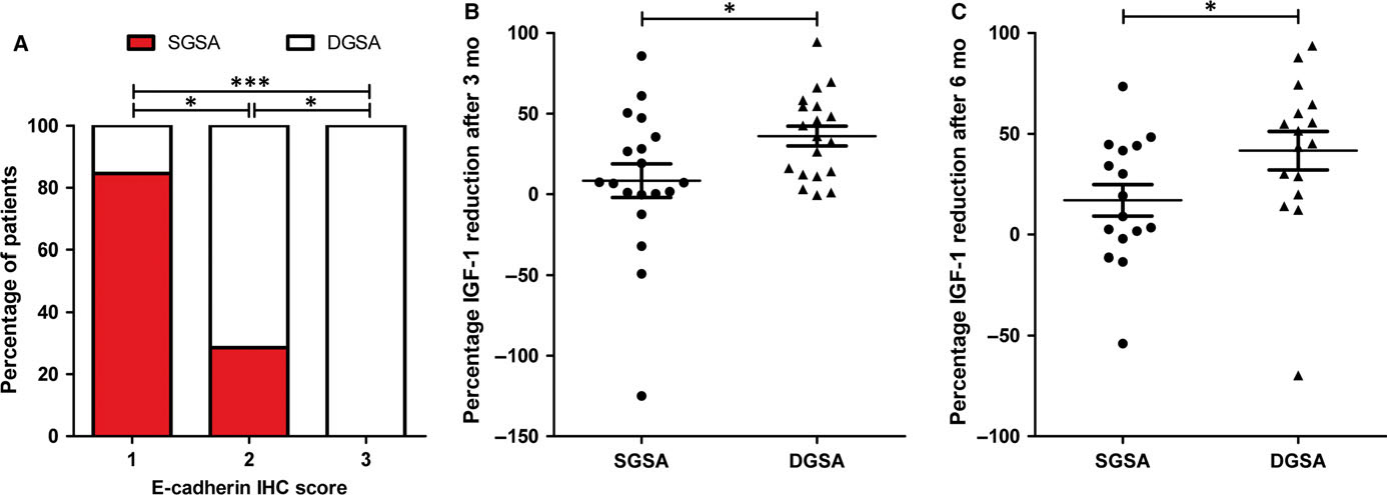
Histological subtypes and response to Somatostatin analogues (SSAs) treatment. A, Percentage of somatropinomas categorized by histology subtype and E‐cadherin immunohistochemistry score. The chi‐square test was used. *P < 0.05; ***P < 0.001. B, Comparison of insulin‐like growth factor 1 (IGF1) per cent reduction after 3 mo of SSAs treatment with the different histological subtypes of GH‐producing tumours. C, Comparison of IGF1 per cent reduction after 6 mo of SSAs treatment with the different histological subtypes of GH‐producing tumours. In (B,C) data points represent values for each individual patient. Mean and SEM are also displayed. The Mann‐Whitney test was used. *P < 0.05

It has been reported that E‐cadherin expression levels differ in GH‐producing tumour histological subtypes.^21^, ^22^, ^23^, ^24^ To confirm this notion in our series of 55 tumours, granulation pattern was examined. Twenty‐four tumours (48%) were SGSAs, 26 tumours (52%) were DGSAs. Histologic subtyping could not be established in five tumours, because of the absence of cytokeratin CAM5.2 immunostaining and these cases were excluded from further analysis. E‐cadherin expression was low or absent in most of SGSAs (Figure 3A) while most of DGSAs displayed strong E‐cadherin expression (score 2 or 3) (Figure 3A). We analysed whether histological subtypes of GH‐producing tumours displayed differences in the response to SSAs treatment. The IGF1 per cent reduction at both 3 (Figure 3B; *P* = 0.027) and 6 months after treatment (Figure 3C; *P* = 0.015) was lower in SGSAs compared to DGSAs. Only one SGSAs (of 16) was responder at 6 months while more than half (nine of 16) of DGSAs were responders (*P* = 0.006). At 3 months of treatment, no significant differences in terms of responders were observed between SGSAs and DGSAs.

## 4 DISCUSSION

In this study, E‐cadherin expression was assessed by IHC in 55 acromegaly patients with GH‐producing tumours, using an automated system and an E‐cadherin antibody widely used in diagnostic pathology. In previous studies on pituitary tumours, antibodies directed against the intracellular or extracellular domain of E‐cadherin have been used.^14^, ^17^ Here, we selected the clone 36 mouse monoclonal E‐cadherin antibody directed against the intracellular domain of the protein; first because this antibody has been increasingly used in recent years for diagnostic pathology, particularly in breast and lung tumours.^16^ Also, because antibodies directed against the extracellular domain of E‐cadherin have previously failed to reveal relevant associations with characteristics of clinical interest in acromegaly.^14^ The reasons for this difference between antibodies that target the extracellular and the intracellular domain of E‐cadherin is unclear but it might be because of differential cleavage and/or internalization of the protein domains^14^ that it might also reflect the differential function of both domains.^35^ Thus, based on the E‐cadherin IHC pattern observed with the selected antibody, three types of somatropinomas could be easily identified in our study: tumours with very low or total absence of E‐cadherin expression (score 1), tumours with a mild to moderate number of cells with E‐cadherin membranous accumulation (score 2, <50% of the tumour cells) and tumours with ample membranous accumulation (score 3, more than 50% of the cells). In our series of GH‐producing pituitary tumours, only membranous localization of E‐cadherin was observed and, in contrast with previous studies,^14^, ^17^ no nuclear accumulation of E‐cadherin could be detected. This apparent discrepancy could be because of the specific E‐cadherin antibody used.^36^ We observed a similar proportion of GH‐producing tumours displaying none or very low membranous E‐cadherin immunoreactivity compared to some studies^17^, ^18^ but a higher proportion compared to other studies.^19^, ^23^, ^37^ These discordant results could be related, at least partly, to differences in patients included in the studies. In this regard, we found a higher number of SGSAs in our series. Nevertheless, our study corroborates the variable expression of E‐cadherin in GH‐producing pituitary tumours previously described in these studies. We found that tumours with elevated E‐cadherin levels (score 3) were markedly smaller compared to tumours with score 1 or 2. This difference in tumour size may explain the marked difference in invasion behavior between tumours with elevated and low or medium E‐cadherin levels. Our results are in agreement with previous studies indicating that lower membranous E‐cadherin levels are associated with aggressive features in pituitary tumours.^14^, ^17^, ^20^

Importantly, our results revealed that loss of membranous E‐cadherin localization is associated with poor response to SSAs in acromegaly patients, in line with a previous study.^14^ IGF1 reduction after 3 and 6 months of SSAs treatment was markedly lower in GH‐secreting tumours with E‐cadherin IHC score 1 compared to tumours with scores 2 and 3. Furthermore, only 2 of the patients with tumours with score 1 (of 20) displayed an IGF1 decrease higher than 50% at 3 months of SSAs treatment, and only 1 of 17 at 6 months after treatment. In contrast, around half of tumours with scores 2 and 3 were non‐responders. Interestingly, no differences in IGF1 per cent reduction were observed between tumours with score 2 and 3, despite the marked differences in E‐cadherin accumulation. Collectively, these results would be consistent with the notion that E‐cadherin membranous localization is a permissive, but not sufficient, factor for the efficient response to SSAs treatment in acromegaly patients.

Tumour expression of SSTR2 seems to be the most consistent marker determining the response to SSAs in acromegaly (recently reviewed in 3 and 26). Thus, tumours with low SSTR2 expression commonly display poor response to SSAs treatment. In support of this, we recently evaluated systematically the expression of SSTRs and DRDs in somatotropinomas by qPCR and found an association between the response to SSAs treatment and SSTR2 but also with SSTR1, DRD4 and DRD5 expression.^13^ However, we did not find here an association between E‐cadherin IHC score and the expression of these receptors. Similarly, there was no association between E‐cadherin and SSTR2 scores when both were evaluated by IHC. Of note, low E‐cadherin levels were associated with poor response to SSAs treatment, even in patients with high SSTR2 levels. Thus, our results suggest that E‐cadherin and SSTR2 might be, at least in part, two independent regulators (and markers) of the response to SSAs. At variance with our results, a previous report has described a direct correlation between E‐cadherin and SSTR2 expression in GH‐secreting tumours.^14^ The reasons for this apparent discrepancy are not completely clear but may relate to differences in the patients included in the studies. Thus, all the patients included in our study received preoperative treatment with SSAs (unlike those patients in^14^), and previous studies have suggested that preoperative SSAs treatment may lead to reduced SSTR2 expression.^10^, ^38^ Nevertheless, our previous analysis of this group of tumours has confirmed that SSTR2 expression adequately discriminates between good and poor responders to SSA treatment^13^ results that compare well with those reported in SSA‐naive patients, thus arguing against a substantial impact of SSAs preoperative treatment on SSTR2 expression. Notably, preoperative treatment of GH‐secreting tumours with SSAs has also been shown to be associated with lower E‐cadherin levels. However, this effect was only observed when E‐cadherin expression was measured by Western blotting, not when evaluated by IHC.^14^ Hence, as we have evaluated E‐cadherin expression by IHC, it is not expected that preoperative SSA treatment might have markedly impacted our results regarding E‐cadherin expression levels. Thus, the potential relationship, or lack thereof, between E‐cadherin and SSTR2 in the context of patient response to SSAs treatment remains unclear and clearly deserves further investigation.

Intriguingly, we observed a negative association between E‐cadherin and SSTR5 expression in GH‐producing tumours. Tumours with low E‐cadherin score expressed higher levels of SSTR5 while tumours with high E‐cadherin score displayed lower SSTR5 protein levels. This unexpected association may be related to the tumour histological subtypes. In our study, most of the GH‐producing tumours with absent E‐cadherin expression were SGSAs, and it has been reported that SSTR5 expression is higher in SGSAs compared to DGSAs.^39^ However, it is important to note that not all studies have observed a difference in SSTR5 expression between SGSAs and DGSAs.^22^, ^40^ Finally, while the relationship between E‐cadherin and SSTR5 expression is poorly know, it is worth noting that the expression of SSTR5 and some of its truncated variants comprises the only known markers among SSTRs for worse patient response to SSAs.^26^, ^31^ Hence, it seems that the potential connection negative linking E‐cadherin and SSTR5 expression (and, perhaps, function) deserves to be explored in more detail.

As previously reported,^21^, ^22^, ^23^, ^24^, ^25^ we found E‐cadherin expression levels are associated with GH‐producing tumour histological subtypes. SGSAs tumours displayed low E‐cadherin levels while DGSAs tumour showed high E‐cadherin levels. Our results confirm and further expand previous data demonstrating that SGSAs exhibit a poor response to SSAs treatment.^21^, ^41^ Indeed, we observed a marked difference in response to SSAs treatment according to the histological subtype; however, this was observed only at 6 months after treatment. Thus, and at least in our group of tumours, we found the E‐cadherin expression was a better biomarker of response to SSAs than histological classification of tumour granulation pattern.

In conclusion, considering that low E‐cadherin levels correlate with poor response to SSAs in GH‐producing tumours, it seems plausible that E‐cadherin may contribute to mediate SSAs effects in these tumours. Accordingly, IHC assessment of E‐cadherin might be useful in categorizing acromegaly patients based on the response to SSAs.

## CONFLICT OF INTEREST

The authors confirm that there are no conflicts of interest.
